# The best of both worlds: a hybrid approach for optimal pre- and intraoperative identification of sentinel lymph nodes

**DOI:** 10.1007/s00259-018-4028-x

**Published:** 2018-04-25

**Authors:** G. H. KleinJan, E. van Werkhoven, N. S. van den Berg, M. B. Karakullukcu, H. J. M. A. A. Zijlmans, J. A. van der Hage, B. A. van de Wiel, T. Buckle, W. M. C. Klop, S. Horenblas, R. A. Valdés Olmos, H. G. van der Poel, F. W. B. van Leeuwen

**Affiliations:** 1 Interventional Molecular Imaging Laboratory, Department of Radiology, Leiden University Medical Center, Albinusdreef 2 (C2-S zone), PO BOX 9600, 2300 RC Leiden, The Netherlands; 2 Department of Nuclear Medicine, The Netherlands Cancer Institute – Antoni van Leeuwenhoek Hospital, Amsterdam, The Netherlands; 3 Department of Urology, The Netherlands Cancer Institute – Antoni van Leeuwenhoek Hospital, Amsterdam, The Netherlands; 4 Department of Biostatistics, The Netherlands Cancer Institute – Antoni van Leeuwenhoek Hospital, Amsterdam, The Netherlands; 5 Department of Head and Neck Surgery and Oncology, The Netherlands Cancer Institute – Antoni van Leeuwenhoek Hospital, Amsterdam, The Netherlands; 6 Department of Gynecology, The Netherlands Cancer Institute – Antoni van Leeuwenhoek Hospital, Amsterdam, The Netherlands; 7 Department of Surgery, The Netherlands Cancer Institute – Antoni van Leeuwenhoek Hospital, Amsterdam, The Netherlands; 8 Department of Pathology, The Netherlands Cancer Institute – Antoni van Leeuwenhoek Hospital, Amsterdam, The Netherlands

**Keywords:** Hybrid, Fluorescence imaging, Nuclear medicine, Sentinel lymph node biopsy, Image-guided surgery

## Abstract

**Purpose:**

Hybrid image-guided surgery technologies such as combined radio- and fluorescence-guidance are increasingly gaining interest, but their added value still needs to be proven. In order to evaluate if and how fluorescence-guidance can help realize improvements beyond the current state-of-the-art in sentinel node (SN) biopsy procedures, use of the hybrid tracer indocyanine green (ICG)-^99m^Tc-nancolloid was evaluated in a large cohort of patients.

**Patients and methods:**

A prospective trial was conducted (n = 501 procedures) in a heterogeneous cohort of 495 patients with different malignancies (skin malignancies, oral cavity cancer, penile cancer, prostate cancer and vulva cancer). After injection of ICG-^99m^Tc-nanocolloid, SNs were preoperatively identified based on lymphoscintigraphy and SPECT/CT. Intraoperatively, SNs were pursued via gamma tracing, visual identification (blue dye) and/or near-infrared fluorescence imaging during either open surgical procedures (head and neck, penile, vulvar cancer and melanoma) or robot assisted laparoscopic surgery (prostate cancer). As the patients acted as their own control, use of hybrid guidance could be compared to conventional radioguidance and the use of blue dye (*n* = 300). This was based on reported surgical complications, overall survival, LN recurrence free survival, and false negative rates (FNR).

**Results:**

A total of 1,327 SN-related hotspots were identified on 501 preoperative SPECT/CT scans. Intraoperatively, a total number of 1,643 SNs were identified based on the combination of gamma-tracing (>98%) and fluorescence-guidance (>95%). In patients wherein blue dye was used (*n* = 300) fluorescence-based SN detection was superior over visual blue dye-based detection (22–78%). No adverse effects related to the use of the hybrid tracer or the fluorescence-guidance procedure were found and outcome values were not negatively influenced.

**Conclusion:**

With ICG-^99m^Tc-nanocolloid, the SN biopsy procedure has become more accurate and independent of the use of blue dye. With that, the procedure has evolved to be universal for different malignancies and anatomical locations.

**Electronic supplementary material:**

The online version of this article (10.1007/s00259-018-4028-x) contains supplementary material, which is available to authorized users.

## Introduction

Sentinel node (SN) biopsy is a routine procedure in the management of breast cancer and melanoma, where it allows for loco-regional staging of lymph nodes (LNs) [1, 2]. This procedure is also more and more applied as a staging-tool for other cancers, e.g. prostate and penile cancer [3, 4]. For the identification of SNs, targeting nanoparticles entitled radiocolloids (e.g. ^99m^Tc-nanocolloid) are considered the standard. The migration kinetics and accumulation of radiocolloids in individual lymphatic basins can be mapped in a non-invasive manner using lymphoscintigraphy and SPECT/CT [5]. For intraoperative tracing of radiocolloid containing SNs, a portable gamma camera or more common a gamma probe can be used [6].

For the surgeons convenience, however, the radioguidance approach is often strengthened by optical guidance in the form of blue dye [7]. Unfortunately, blue dye also has some short-comings, the main ones being the staining of the injection site, allergic reactions and the limited degree of nodal identification [8]. The last is a result of the lack of SNs specificity of this lymphangiographic agent and the limited sensitivity of this light-absorbance based detection method. Almost two decades ago, the fluorescent dye indocyanine green (ICG) was introduced as an alternative lymphangiographic dye [9]. At high concentrations ICG is green in color, but at low concentrations it can only be detected using dedicated near-infrared fluorescence cameras (maximum emission intensity = 820 nm) [10]. One of the advantages, compared to blue dye is the superior penetration depth of the fluorescence emission (up to 1 cm). Nevertheless, this penetration depth is vastly inferior to the >10 cm penetration of a radioactive signature of ^99m^Tc [11]. Furthermore, as a result of its molecular size, ICG still shares a shortcoming with blue dye, namely, an unrestricted lymphatic migratory pattern and with that a lack of specificity for SNs compared to higher echelon nodes. This same feature also induces spillages during surgical manipulations of the tissue.

In an ideal situation, the best features related to both the radioguided surgery and optical guidance procedures would be integrated, meaning SN specificity should be combined with sensitive intraoperative optical guidance. Such a best-of-both-worlds scenario was realized following the development of the hybrid tracer, ICG-^99m^Tc-nanocolloid and its clinical translation in 2009 [12]. In various malignancies this hybrid tracer was shown to allow: (1) non-invasive preoperative localization of the SNs using scintigraphy and or SPECT/CT, (2) intraoperative rough localization based on the gamma signal and/or using radioguidance based navigation approaches, and (3) detailed superficial (< 1 cm) intraoperative fluorescence guidance [13–16].

**Fig. 1:**
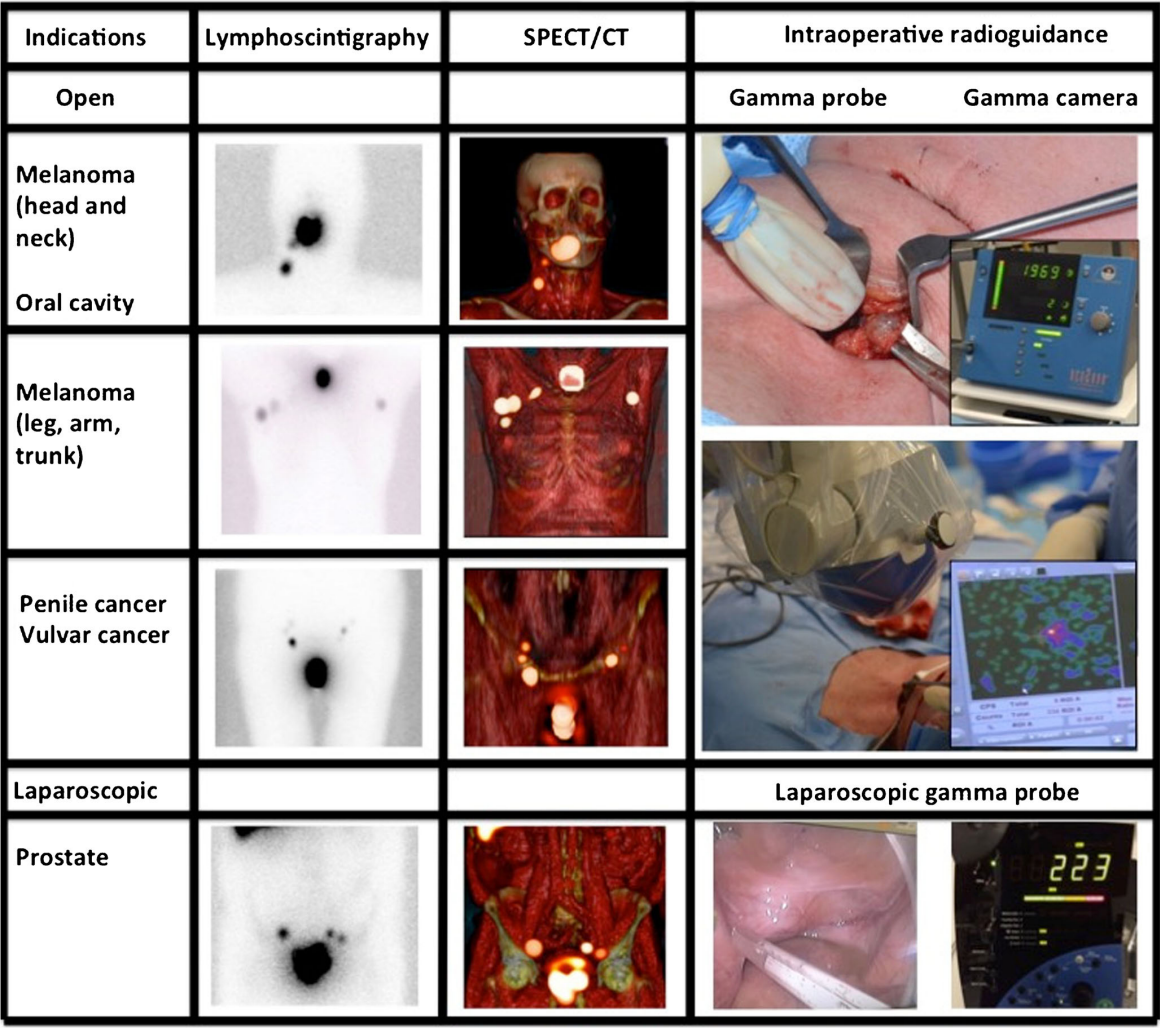
Radioguidance enabled by the hybrid tracer*.* The first three rows presents open surgical procedures (head and neck area, trunk and groin), while the last row presents a laparoscopic procedure (pelvis). The second and third column display examples of preoperative lymphoscintigraphy and SPECT/CT illustrating drainage to the neck, axilla, groin and pelvis. The last column gives an indication of the radioguidance technologies used intraoperatively

Promising new surgical guidance technologies using radio- and fluorescence-imaging are gaining interest from a scientific, medical and industrial perspective. However, translation of such technologies generally has been restricted to first-in-human trials with small patient groups and in specialized academic hospitals [11]. Internal controls and follow up of patients and outcome data for larger and more heterogeneous patient groups are essential to benchmark new technologies and assess their added value over the state-of-the-art. This study presents the validation of the ICG-^99m^Tc-nanocolloid based hybrid SN procedure in a relatively large (495 patients) and heterogeneous patient group (skin malignancies, oral cavity cancer, penile cancer, prostate cancer and vulva cancer) and includes both follow-up and outcome data. By relating the hybrid procedure to the traditional radioguidance and blue dye approaches, in the same patient (Fig. 1), the added value of the new technology was investigated/determined.

## Methods

Between March 2010 and January 2016, 495 patients were prospectively included in 501 SN procedures (some patients were scheduled for re-resections including SN procedure). Of this group 234 patients (47%) were previously included in different studies [5, 13–16]. All patients were operated at the Netherlands Cancer Institute – Antoni van Leeuwenhoek Hospital. Approval for these studies was obtained from the local institution review board and the trials was registered under reference NL26699.031.09 and NL40636.031.12. Following initial examination in a trail context, off-label use of the hybrid tracer was approved by the local pharmacy (approval received in November 2012). This was only allowed in indications were the SN procedure is considered standard of care in our hospital.

### Patients

All included patients were ≥ 18 years of age and had a histologically proven malignancy (See Supplemental information (SI), Table SI1). All patients were regional clinically node-negative (cN0) as defined by palpation and ultrasound-guided fine-needle aspiration cytology. Patients with squamous cell carcinoma (SCC) of the penis and vulva were also eligible when cN1 was on the contralateral side. All patients underwent SN biopsy and subsequent treatment of the primary tumor site.

The fully prepared hybrid tracer (ICG-^99m^Tc-nanocolloid) was commercially obtained from the Dutch GE Healthcare radiopharmacy (Leiderdorp, The Netherlands). Injection procedure, preoperative imaging procedure, surgical procedures and pathological examination have been reported previously and are explained in more detail in the supporting information section [5, 13–16].

The order wherein the surgical guidance technologies were used were in agreement with a previously described protocol [14]. In brief, prior to the start of SN resection, in open procedures a portable gamma camera (Sentinella; Oncovision, Valencia, Spain) and gamma probe (Neoprobe; Johnson & Johnson Medical, Hamburg, Germany) were used to assess the distribution of the SNs in the surgical field. This was followed by a directed incision and the application of SNs using a (laparoscopic) gamma probe (Europrobe, Strassbourg, France). When blue dye was administered, blue coloration was used to identify the location of tumor draining lymphatic vessels and blue dye containing SNs. Finally, fluorescence imaging was performed to confirm the location of the SNs in high resolution and to support their surgical resection (PhotoDynamic Eye; Hamamatsu Photonics, Hamamatsu, Japan or 30° laparoscope HOPKINS II 10 mm; Karl Storz Endoskope GmbH, Tuttlingen, Germany). This order helps identify the added value provided by fluorescence guidance on top of the traditional radio- and blue dye guidance. While this approach ensures optimal patient care, a down side is that it does makes it more complex to isolate the independent value that fluorescence guidance brings to the operating surgeon.

To verify nodal removal, the surgical wound was reexamined using radiotracing/imaging, fluorescence imaging, visual inspection for blue dye and palpation (when possible). When a residual signal was observed at the location of the original SN, this node was considered a missed SN or part of a cluster of multiple adjacent SNs and was removed. In case of clustered nodes, after the operation, the acquired SPECT and CT images were retrospectively evaluated to confirm cluster formation.

## Follow-up

Complication rates within 90 days after surgery were reported following the Clavien-Dindo score and were scored per patient [17].

The overall survival was determined with a maximum follow up of 6 years and an average follow up of 33 months. The overall survival was also determined for different malignancies in different anatomical indications stratified by pN0 R0 and pN+ R0 (N = nodal status and R0 = resection margin negative). This was presented in Kaplan Meier curves (using R statistical software; R Development Core Team, 2008, Vienna, Austria). To yield homogenous patient groups, in the group of skin malignancies only patients with melanoma were included. In the penile cancer group only the SCC patients were included and this was also the case for the patients with tumors located on the vulva. For the prostate cancer patients we reported the biochemical recurrence rate (BCR)-free (prostate specific antigen (PSA) >0.01 ng/ml) survival as indication of disease-free survival [5].

False negative patients were defined as patients with LN recurrence during follow up and negative SN in the originally explored LN basin and negative non-SN at time of primary surgery [18]. Patients with synchronous primary tumor recurrence and LN recurrence were excluded from this calculation. Because FNR calculations are based on tumor containing SNs that were missed during resection (note: no attempts were made to resect draining lymphatic vessels), patients that presented metastases within lymphatic vessels (so-called in-transit metastases) on follow-up were excluded from the FNR calculation.

To allow for comparison between the group where blue dye was used and only the hybrid tracer was used, the LN recurrences were determined on a per patient basis. For the SN penis and vulva procedures the FNR per groin was also reported. When applicable, the FNR rates were also reported without inclusion of the first 15 procedures, which were considered as an initial learning curve with the technology.

## Statistical analysis

The intraoperative SN detection rates were calculated as percentages of SNs. Fluorescence detection was compared to blue detection and gamma detection for the overall group, and per indication. Associations of fluorescence detection in vivo and ex vivo with BMI of the patient were investigated with logistic regression, using the Huber-White method to adjust the variance-covariance matrix to correct for correlated responses from nodes clustered within the same patient (using R function robcov from package rms) [19]. A 95% confidence interval (CI) was given, and a *p*-value ≤0.05 was considered significant.

Wilcoxon’s rank sum tests were performed to test associations between whether primary tumor site removal was before or after SN biopsy and SN detection rates. The same test was used to assess associations of a 1- or 2-day protocol with the SN detection rates and the gamma counts measured by the gamma probe. A log-rank test was used to analyze the statistical differences between the Kaplan Meier curves.

## Results

### Preoperative SN mapping via nuclear imaging

In the SI section, the preoperative-imaging findings are described. In total 1,327 SN-related hotspots were identified in these images.

### Intraoperative SN identification rates enabled by surgical guidance modalities

**Fig. 2:**
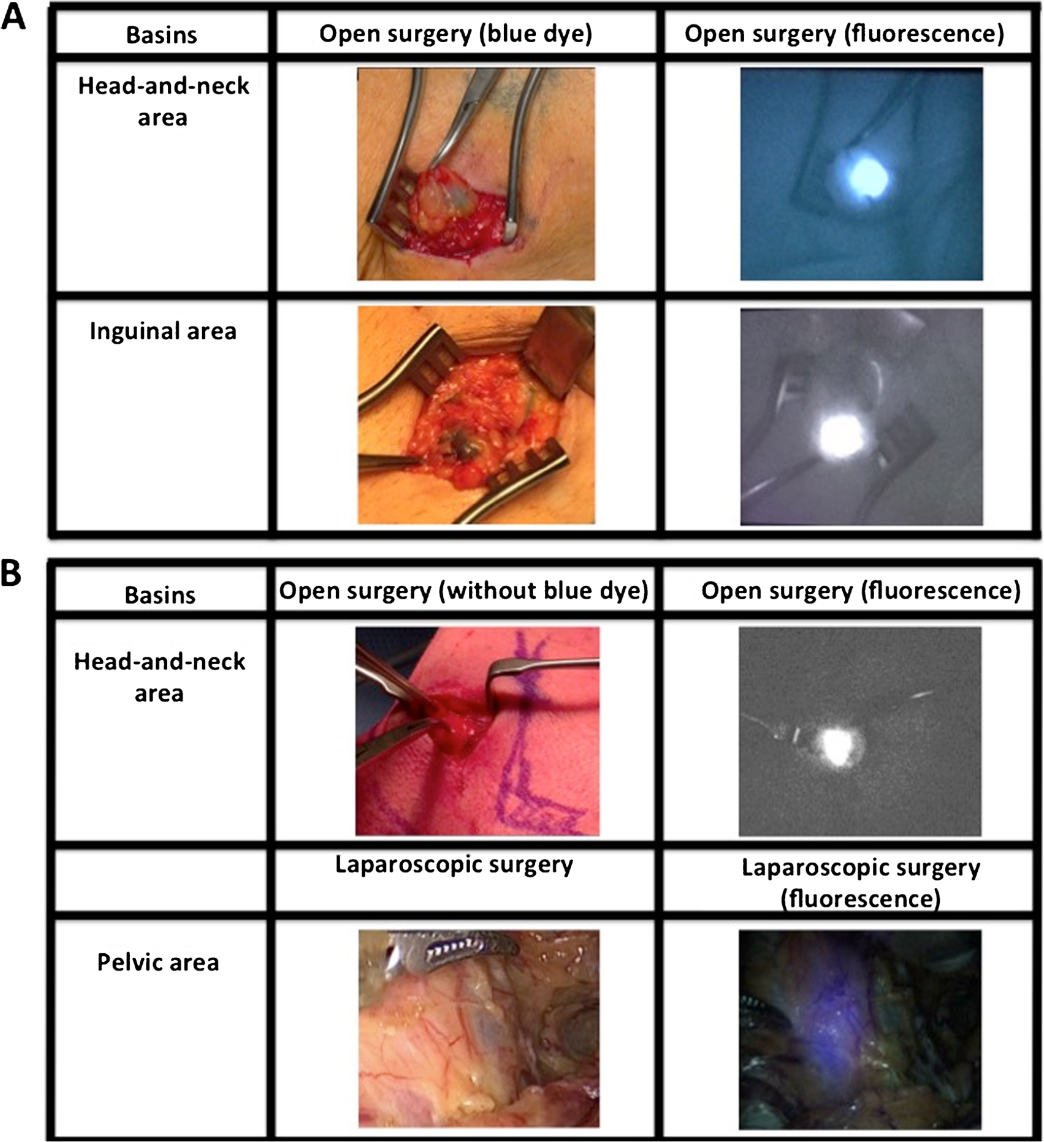
Optical guidance enabled by the hybrid tracer and blue dye. **a** Typical examples of procedures combining use of blue dye and the hybrid tracer ICG-^99m^Tc-nanocolloid in the head-and-neck region and groin. **b** Typical examples of procedures using only the hybrid tracer for optical guidance (head-and-neck and pelvic region). The second column demonstrates what is seen by eye, while the third column provides insight into the signal obtained via fluorescence imaging with a (laparoscopic) near-infrared dedicated fluorescence camera

The intraoperative detection rates using radioguidance, fluorescence and blue dye are summarized in Fig. 2, Table 1 and Table SI2. In total 1,643 SNs specimens (containing 1,938 SNs) were surgically removed.

**Table 1:** Fluorescence-, blue- and radioactivity-based detection rate per indication (see also Fig. 2)

Parameter	Skin malignancies body	Head-and-neck skin malignancies		Oral cavity		Penis	Prostate	Vulva	Total
In vivo detection rates									
Blue used	Yes	Yes	No	Yes	No	Yes	No	Yes	Total
No optical identification (SNs)	2 (2%)	1 (1%)	15 (4%)	–	7 (4%)	9 (1%)	28 (22%)	2 (3%)	64 (4%)
Blue only SNs	1 (1%)	0	–	0	–	6 (1%)	–	0	7 (0%)
Fluorescent only^a^ SNs	37 (28%)	73 (68%)	334 (89%)	3 (50%)	149 (78%)	244 (38%)	99 (78%)	21 (34%)	960 (58%)
Fluorescent^a^ and blue SNs	88 (67%)	24 (22%)	–	0	–	272 (42%)	–	34 (55%)	418 (25%)
Total fluorescent SNs	125 (97%)	97 (99%)	334 (96%)	3 (100%)	149 (96%)	516 (97%)	99 (78%)	55 (96%)	1378 (95%)
SNs not evaluated for staining	3 (2%)	10 (9%)	25 (7%)	3 (50%)	36 (19%)	112 (17%)	0	5	194
Total radioactive^a^ SNs	131 (100%)	108 (100%)	356 (95%)	5 (83%)	134 (70%)	622 (97%)	82 (65%)	61 (98%)	1499 (91%)
SNs not evaluated for radioactivity	0	0	8	0	43	8	45	1	105
Total (in vivo and ex vivo^b^ combined)									
Blue used	Yes	Yes	No	Yes	No	Yes	No	Yes	
No optical identification (SNs)	0	1 (1%)	3 (1%)	0	3 (2%)	5 (1%)	1 (1%)	1 (2%)	14 (1%)
Blue only SNs	0	0	–	0	–	9 (1%)	–	0	9 (1%)
Fluorescent^a^ only SNs	36 (27%)	81 (75%)	368 (99%)	5 (83%)	184 (98%)	284 (44%)	126 (99%)	22 (35%)	1106 (67%)
Fluorescent^a^ and blue SNs	95 (73%)	26 (24%)	1 (0%)		–	323 (50%)	–	39 (63%)	484 (29%)
Total fluorescent SNs	131 (100%)	107 (99%)	368 (99%)	5 (100%)	184 (98%)	607 (98%)	126 (99%)	61 (98%)	1589 (99%)
SNs not evaluated for staining	0	0	2 (1%)	1 (17%)	5 (3%)	22 (3%)	0	0	30 (2%)
Total radioactive^a^ SNs	131 (100%)	108 (100%)	373 (100%)	6 (100%)	180 (98%)	625 (98%)	127 (100%)	61 (100%)	1611 (99%)
SNs not evaluated for radioactivity	0	0	0	0	9	6	0	1	16

*SN* sentinel node

^a^Due to the hybrid nature of the tracer the fluorescence and radioactive signatures are directly related

^b^Ex vivo validation of the fluorescence signal because of tissue attenuation whereby the in vivo detection of the fluorescent signal could be hampered. Ex vivo the SNs are more exposed and as such the fluorescence detection increases

Of the SNs that were surgically removed, 99% (in vivo and ex vivo combined) could be detected using gamma tracing. This was the leading guidance technology and provided a benchmark for both blue dye and fluorescence. Overall, using the hybrid tracers’ fluorescence signature, the SNs could be optically identified in >95% (in vivo and ex vivo combined) of the cases (combined in vivo and ex vivo examination in the surgical theater). The anatomy of the basin in which the SNs were situated did not influence these find rates (Fig. 2). The hybrid tracer nodal dissections did not suffer from contamination of the surgical field as a result of tracer leakage from the lymphatic system. Of the SN specimens 12% (194/1643) were superficially located and could be resected without the use of the fluorescence signature (note: these SNs did clearly express a fluorescent signature). Despite the slightly lower detection rate > 95% vs. 99%, in more complex anatomical locations, e.g. the head-and-neck or pelvic area, the fluorescence signature of the hybrid tracer was valued for its superior spatial resolution and the ability to visualize the SNs with the anatomical context (Figure SI2). Potential damage to surrounding structures withheld the surgeon from resection in 2.2% (29/1327) of the SN-related hotspots identified on SPECT/CT, e.g. in the neck or pararectal space. In these cases, despite a clear gamma read-out of the same tracer, the inability to detect a fluorescence signal indicated the SNs were located >0.5–1 cm deep within the tissue. This fluorescence-based depth-estimation helped improve a benefit/risk balance on which the decision was made to abandon further dissection in these areas. In the head and neck, groin and pelvis, this feature of the hybrid tracer made the operating surgeon generally rely more on intraoperative fluorescence guidance than on intraoperative radioguidance. In eight patients (1.6% of total) radioguidance could not distinguish the SN from the injection site, yet these SNs were preoperatively identified by SPECT/CT and could readily be resected under fluorescence guidance. When SNs were located deeper than 0.5–1 cm from the tissue surface, fluorescence guidance was unreliable and a combination of the SPECT/CT findings and the radioactive signature of the hybrid tracer were instrumental for the nodal localization.

**Fig. 3:**
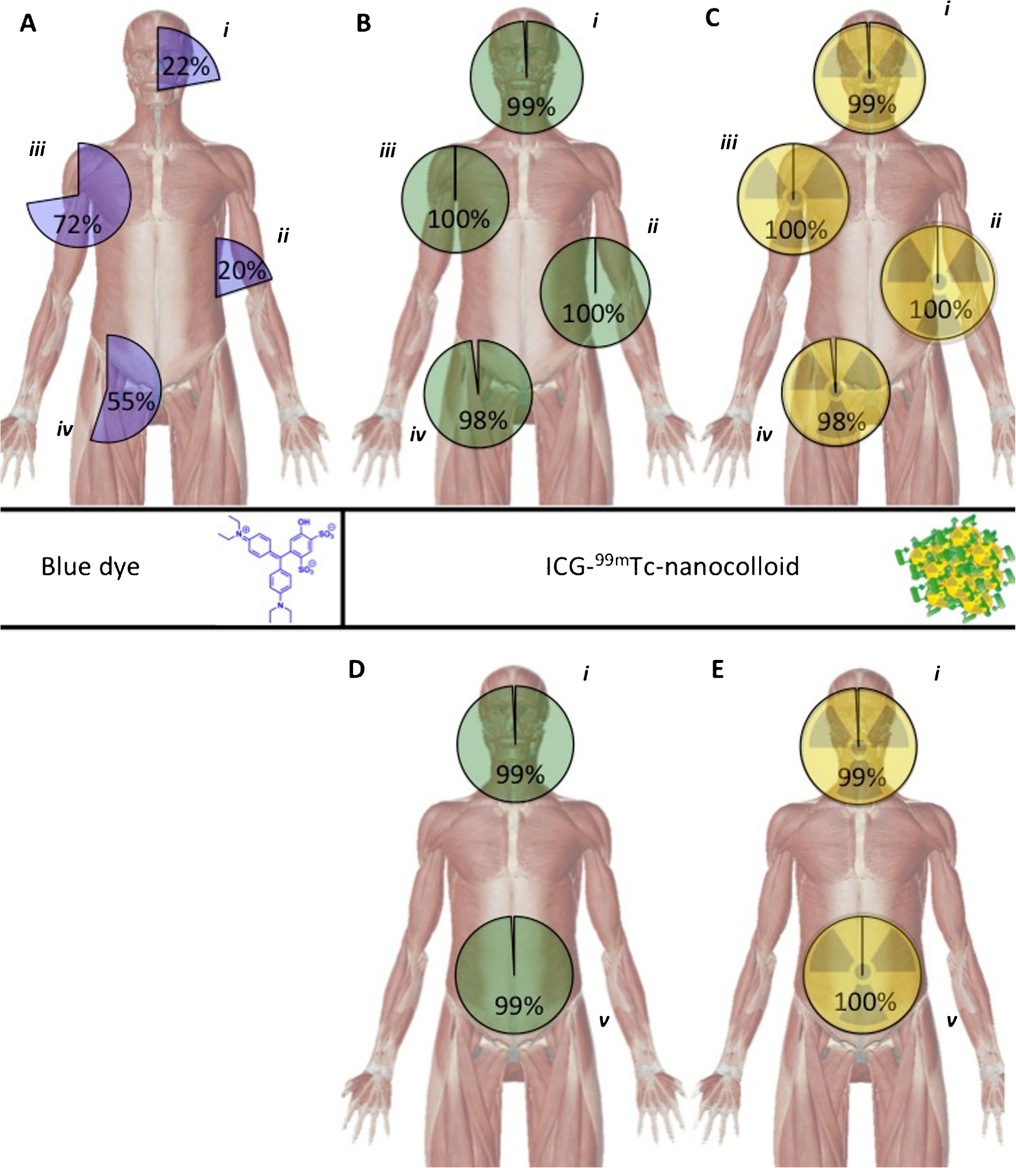
Intraoperative detection rates for the different surgical guidance modalities used. **a** Blue dye-based SN identification percentages in patients who received blue dye subsequently to the hybrid tracer (total *n* = 300). **b** Fluorescence-based SN identification percentages (total *n* = 501). **c** Radioactivity-based SN identification percentages (total *n* = 501). **d** and **e** respectively depict the fluorescence-based and radioactivity based SN identification percentages in the patient groups that did not receive blue dye (total *n* = 201). Detection rates are provided for different regions: *i*) head and neck area, *ii*) arm and trunk, *iii*) axilla, *iv*) inguinal area and *v*) pelvic region

For the procedures where blue dye was used, a wide variation in blue stained SNs was observed in the SNs (20–72%; *n* = 300; 950 SNs; Fig. 3). Blue dye was most helpful in the axilla (72%), but proved to be of limited value in the head-and-neck area (23%) as well as the arm and shoulder (20%). Blue dye-based find rates were significantly poor compared to those obtained with the fluorescent signature of the hybrid tracer (*p*-value <0.001). Merely 1% (9 SNs; in vivo and ex vivo combined) was dissected based on blue coloration only (and SI Table 3). Hence the availability of the hybrid tracer (meaning the availability of a fluorescence and/or a radioactive signature) in the SNs was considered to provide superior >95% guidance compared to blue dye.

The relation between pre-operative and intraoperative SN find rates, the influence of the surgical resection order (see Figure SI1) and BMI (see Figure SI2) on the surgical guidance procedure are described in the SI section.

### Pathological evaluation

From the excised 1,643 SN specimens (containing single LNs or LN clusters), 1938 LNs were harvested at pathology. In Table SI2 the percentage of tumor positive SNs is reported. In the SI a further description of the tumor find rate (TFR) is provided.

### Follow-up

The complication grade score according to Clavien-Dindo ranged from I–V in this study cohort and was highest in the group of penile cancer patients (Table SI4). In none of the 495 patients did adverse effects occur that could be related to the use of the hybrid tracer or blue dye. Also, there was no correlation seen between the removal of additional SNs and occurrence of procedure-related complications (*p*-value 0.478). The overall survival results are provided in the SI section (Figure SI3).

**Fig. 4:**
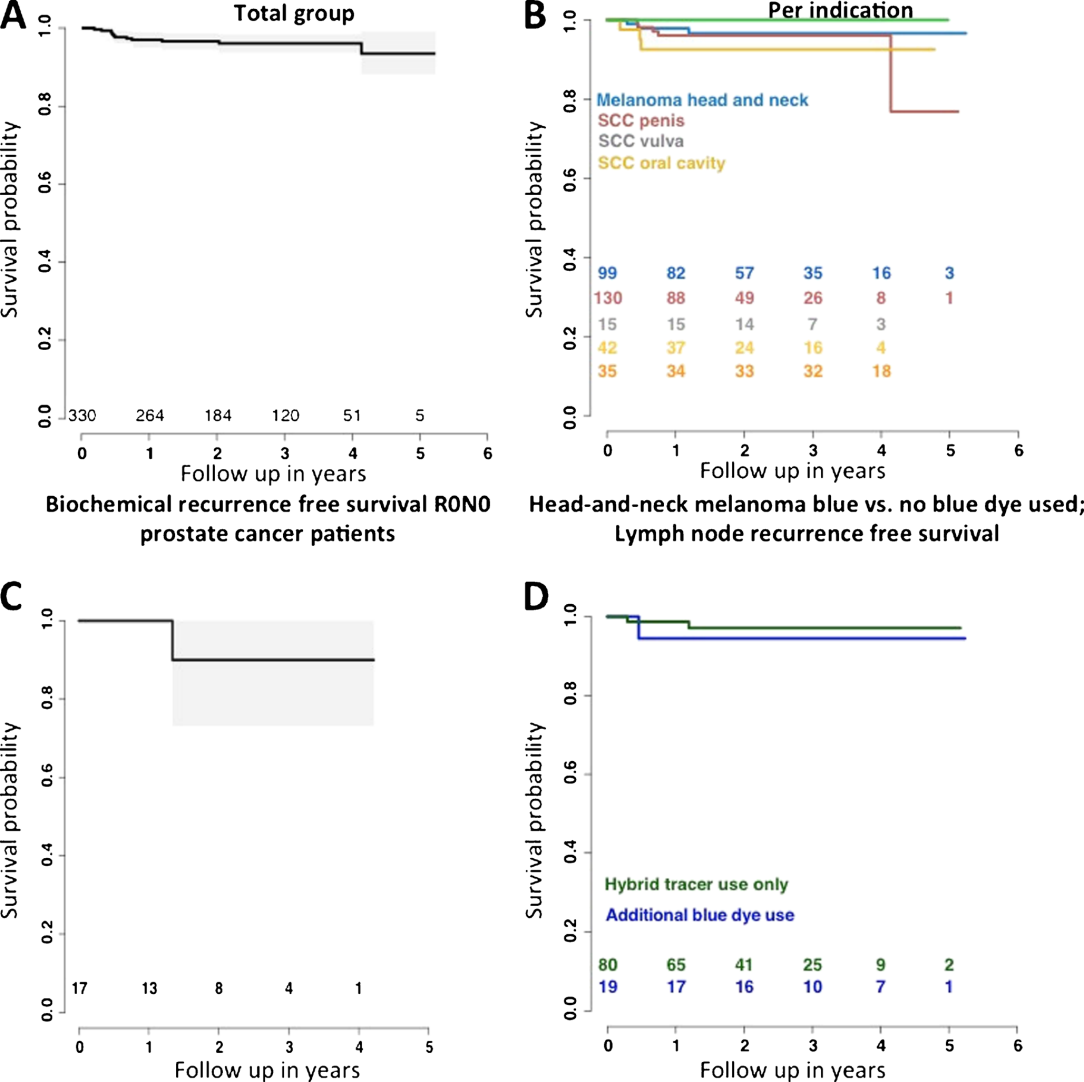
Lymph node- and biochemical recurrence curves. In **a** and **b** the LN recurrence curves are shown for the total group and per indication, respectively. **c** Biochemical recurrence free survival of pN0 R0 prostate cancer patients. To relate outcome to the use of the hybrid tracer with or without the use of blue dye, recurrence rates are shown for the head melanoma group. **d** LN recurrence rates for R0N0 head-and-neck melanoma patients (*p* = 0.29)

The average follow up in this study was limited to 33 months. In pN0 R0 patients (*n* = 321; 65%), the LN recurrence-free survival varied from 93 to 100% for the various indications, with an average of 93% at two-year follow-up (Fig. 4a, b). In the prostate cancer patients, a BCR-free survival of 90% (CI 73–100%) was found at 2-year follow-up (Fig. 4c). No LN recurrences occurred in patients with melanoma on the body or vulvar cancer. In the penile cancer patients, the LN recurrence-free survival rate was the lowest (77%) at 5-year follow-up.

In a relatively small group of pN0 R0 patients with head-and-neck melanoma, wherein the hybrid tracer was used in combination with (*n* = 19) or without (*n* = 80) blue dye, we were able to study if use of blue dye had an influence on LN recurrence-free survival. The group wherein the hybrid tracer alone was used had a 97% (CI 93%-100%) LN recurrence-free survival probability, while the group with the hybrid tracer in combination with blue dye had a slightly lower LN recurrence-free survival of 94% (CI 84-100%). Both groups were comparable for T-stage. These findings indicate that use of blue dye next to the hybrid tracer does not lead to a better prognosis (Fig. 4d).

In the overall population, ten isolated LN recurrences (2%) were reported, yielding a combined FNR of 14% (see SI Table 4 for the FNRs of the individual procedures). Interestingly, false negative findings (during follow up) of oral cavity patients occurred in the first 15 cases. After this learning curve, the combined FNR decreases to 10.3% (Table SI4). A low FNR was found in the melanoma body group (0%) and a modest FNR (7%) in the melanoma head-and-neck group. The obtained values were lower than the weighted literature FNRs for head-and-neck melanoma, which averaged 12.5% (range 0–34%) [20]. The FNR in oral cavity cancer (22%) was in line with the range previously reported (9–29%) [21, 22]. In the penile cancer group the FNR was 16%, which was lower than the initial report of the sentinel node biopsy for penile cancer (22%) described by Tanis et al. [23]. The FNR rate for penile cancer further dropped to 14% following exclusion of the re-sentinel node procedures, which indicates the 16 repeat SN procedures in patients with local recurrence had a negative influence on the FNR (Table SI4). The 0% FNR we found for the vulvar cancer patients is in line with the report of Van der Zee et al. (2.3–3%) [24]. The FNR of the SN procedure in the prostate cancer group compared to the extended pelvic LN dissection (ePLND) was 11.3%, again comparable to earlier reports [4].

## Discussion

The present longitudinal study of a 495-patient cohort indicates that the generation of a hybrid set-up that includes fluorescence guidance in an otherwise standard SN-procedure creates value for the operating surgeons by: (1) improving optical guidance compared to blue dye (>95% vs. 20–72%, respectively (Table 1; *p*-value < 0.001), (2) providing benefit over radiotracing as a result of visualizing the SNs within their anatomical context, a feature that was particularly valuable in complex anatomies (95% of the SNs (Table 1)), (3) providing depth estimation (>0.5–1 cm) of the nodal location, which helped prevent surgery related side effects in 2.2% of the SNs and (4) supporting the surgical identification of LNs that reside next to the injection site (1.6% of the SNs). Unlike blue dye, the value of the hybrid tracer was not confined to the anatomical location wherein the procedure was applied, surgical timing, the order of resection (primary tumor vs. SN), or the surgical setting used (open or laparoscopic). Hence, through use of the hybrid tracer, SN procedures can be performed in a uniform manner, allowing expansion of the technology to non-traditional SN indications.

The hybrid approach described provided the ability to perform radioguidance and enhance it by fluorescence imaging of the exact same features. This resulted in a further refinement of the surgical SN identification, e.g. the ability to surgically identify SNs in close location to the injection site helped improve coherence with the surgical findings and the SN-related hotspots detected by preoperative SPECT/CT. The synergistic approach also yielded enhanced intraoperative SN find rates, e.g. during surgery 24% more nodes were identified [13, 16]. In contrast to what has been reported for the use of “free” ICG, the hybrid tracer did not show leakage into the surgical field [25]. Unfortunately, fluorescence imaging (even in the near-infrared region of the light spectrum) suffers from tissue attenuation when compared to radioguidance technologies [11]. The difference between in vivo and ex vivo fluorescence detection rates (Table 1 and Table SI3) underline that radioguidance remains essential for in vivo SN localization and that ex vivo validation remains critical to assess the presence of fluorescence [10]. This effect increases with an increase in BMI (see Figure SI2). Uniquely, in some cases lack of fluorescence detection was considered predictive for the depth in the tissue at which the SN resided.

One aspect that we have not addressed in this patient cohort was the differentiation of SNs from higher echelon nodes via visualization of the afferent lymphatic ducts, a blue dye technique commonly performed by expert surgeons [7]. Although the fluorescence guidance modalities used in this study were able to visualize the afferent lymphatic ducts [16], visualization of these ducts was not relied upon as a routine tool due to the labor intensive exposure of the ducts. We recently reported that lymph duct visualization becomes much more straight forward with next generation fluorescence guidance cameras [26]. When such hardware improvements are integrated, use of the hybrid tracer strengthens a recent statement by Van der Ploeg et al. who suggest that blue dye may potentially be omitted in SN biopsy for melanoma [27]. The LN blue dye dependent free recurrences survival we report for a homogenous head and neck melanoma population (Fig. 4d) also indicates that use of blue dye did not positively influence outcome.

The follow up data provided for the hybrid SN procedure is positive (Fig. 4 and Figure SI3) and an overall false negative rate (FNR) of 10.1% (Table SI4) is acceptable, but from the current data it is not yet possible to conclude whether use of the hybrid tracer improves the oncological outcome. Limiting herein is the average follow up time of 33 months. There also were clear indications that in complex anatomies the procedure includes a learning curve, which was also reported previously [28]. Given the complexity of the diagnostic SN procedure, the limited tumor find-rates, and the dependence on factors that extend beyond the operating theater, e.g. tracer administration and pathological accuracy, we wonder whether expecting such improvements would even be realistic. Following the technical evolution of tracers and surgical guidance modalities, it seems that further refinement of the oncological outcome requires a critical look at the patient inclusion, tracer deposition, and means of pathological evaluation. In this cohort, for example, some patients were staged with a higher T-stage than those described in the treatment guidelines of the different malignancies, which could have negatively influenced the findings (Table SI1). For the tracer deposition, earlier and ongoing studies indicate that its location directly relates to the lymphatic drainage and thus refinements in this area could help improve the ability to detect lymphatic metastasis [29]. Finally, the relation between the FNR and the accuracy of the surgical guidance procedure is not a direct one as the FNR is also highly dependent on the quality of the pathological accuracy, in particular when micro-metastases occur [30]. Hence advances in the pathological examination of SN specimens could also help to improve the procedure.

All surgeons involved in the present study had previous experience with SN-procedures based on radioguidance (^99m^Tc-nanocolloid) and valued the more detailed guidance obtained using ICG-^99m^Tc-nanocolloid. Given the study set-up (order: first radioguidance, e.g. SPECT imaging, use portable gamma camera and gamma tracing, followed by blue dye detection and lastly, fluorescence imaging), the present findings all used radioguidance (the modality used for preoperative imaging, e.g. SPECT/CT and the surgical modality that provides superior (in-depth) detection sensitivity) as reference [8]. Future randomized studies that blind the operating surgeon to different aspects in the hybrid image guidance procedure, e.g. different aspects of radioguidance, could help to provide more detailed insight into the clinical value of the individual components of the technology. It should be noted that the hybrid tracer design solely has the purpose to extend routine radioguidance with fluorescence guidance, rather than replace radioguidance approaches with fluorescence guidance. Moreover, the limited tissue penetration of fluorescence guidance prevents its use for surgical planning [30], a critical aspect in SN procedures. Nevertheless, randomized blinded studies are currently being conducted to accurately determine the dependency of fluorescence guidance on the information provided by nuclear medicine. Alternative features that could be valuable to measure in future trials are operating time and the surgeon’s confidence in decision making.

## Conclusion

This study underlines that the proposed hybrid SN approach, which uses the hybrid tracer ICG-^99m^Tc-nanocolloid, not only provides preoperative SN mapping but also allows for superior optical surgical guidance compared to blue dye.

## Electronic supplementary material

ESM 1 (DOCX 367 kb)
