# Supplementary material for: The best of both worlds: a hybrid approach for optimal pre- and intraoperative identification of sentinel lymph nodes
Source: Eur J Nucl Med Mol Imaging. 2018 Apr 25;45(11):1915–25. doi: 10.1007/s00259-018-4028-x (PMC6132545; doi:10.1007/s00259-018-4028-x)
Supplement: Supplementary file 1 — (DOCX 367 kb) [file 259_2018_4028_MOESM1_ESM.docx]

**Supplemental information**

**The best of both worlds: A hybrid approach for optimal pre- and intraoperative identification of sentinel lymph nodes**

KleinJan GH^1,2,3^, van Werkhoven E^4^, van den Berg NS^1, 3,5^, Karakullukcu MB^5^, Zijlmans HJMAA^6^, van der Hage JA^7^, B.A. van de Wiel, Buckle T^1^, Klop WMC^5^, Horenblas S^3^, Valdés Olmos RA^1,2^, van der Poel HG^3^, van Leeuwen FWB^1,3,5*^

1. Interventional Molecular Imaging Laboratory^1^, Department of Radiology, Leiden University Medical Center, Leiden, the Netherlands;

2. Departments of ^2^Nuclear Medicine, ^3^Urology, ^4^Biostatistics, ^5^Head and neck surgery and oncology, ^6^Gynecology and ^7^Surgery, The Netherlands Cancer Institute – Antoni van Leeuwenhoek Hospital, Amsterdam, the Netherlands.

**Methods**

**Patient characteristics**

**Table SI1. Patient characteristics per anatomical location**

|  | **Skin malignancies body** | **Head-and-neck skin malignancies** | **Oral cavity** | **Penis** | **Prostate** | **Vulva** | **Total** |
| --- | --- | --- | --- | --- | --- | --- | --- |
| **Nr. patients** | 56 | 135 | 51 | 192 | 40 | 21 | **495** |
| **Nr. procedures** | 56 | 135 | 51 | 198 # | 40 | 21 | **501** |
| **Re-sentinel node biopsy** | 0 | 0 | 0 | 14 | 0 | 0 | **14** |
| **Age**  **(median, IQR)** | 52  (42-66) | 61  (50-70) | 61  (56-66) | 67  (59-75) | 64  (61-68) | 70  (54-77) | **64**  **(54-72)** |
| **BMI**  **(median, IQR)** | 25  (22-28) | 26  (24-29) | 25  (23-28) | 27  (25-29) | 26  (25-27) | 26  (22-28) | **26**  **(24-28)** |
|  |  |  |  |  |  |  |  |
| **SCC** | 1  (2%) | 0 | 51  (100%) | 194 (98%) | 0 | 19 (90%) | **265**  **(53%)** |
| **Melanoma** | 54  (96%) | 129  (96%) | 0 | 1  (0.5%) | 0 | 2  (10%) | **186**  **(37%)** |
| **Adenocarcinoma** | 0 | 0 | 0 | 1  (0.5%) | 40  (100%) | 0 | **41**  **(8%)** |
| **MCC** | 1  (2%) | 6 (  4%) | 0 | 0 | 0 | 0 | **7**  **(1%)** |
| **UC** | 0 | 0 | 0 | 1  (0.5%) | 0 | 0 | **1**  **(0%)** |
| **Sarcoma** | 0 | 0 | 0 | 1  (0.5%) | 0 | 0 | **1**  **(0%)** |
|  |  |  |  |  |  |  |  |
| **Clinical TNM** |  |  |  |  |  |  |  |
| **Breslow**  **(median, IQR)** | 2  (1-3) | 2  (1-4) | - | 2  (2-2) | - | 10  (10) | **2**  **(1-4)** |
| **cT1** | 5  (9%) | 15  (11%) | 33  (65%) | 64  (32%) | 10  (25%) | 0 | **127**  **(25%)** |
| **cT2** | 26  (46%) | 48  (36%) | 17  (33%) | 107 (54%) | 30  (75%) | 0 | **228 (**  **46%)** |
| **cT3** | 14  (25%) | 41  (30%) | 1  (2%) | 23  (12%) | 0 | 0 | **79**  **(16%)** |
| **cT4** | 8  (14%) | 27  (20 %) | 0 | 0 | 0 | 1  (5%) | **36**  **(7%)** |
| **FIGO 1** | 0 | 0 | 0 | 0 | 0 | 19 (90%) | **19**  **(4%)** |
| **NA** | 3  (5%) | 4  (3%) | 0 | 4  (2%) | 0 | 1  (5%) | **12**  **(2%)** |
|  |  |  |  |  |  |  |  |
| **cN0** | 56  (100%) | 134  (99%) | 51  (100%) | 182 (92%) | 40  (100%) | 20 (95%) | **483**  **(96%)** |
| **cN1** | 0 | 1  (1%) | 0 | 16  (8%) | 0 | 1  (5%) | **18**  **(14%)** |
|  |  |  |  |  |  |  |  |
| **Pathological TNM** |  |  |  |  |  |  |  |
| **pTx** | 0 | 0 | 0 | 8  (4%) | 0 | 0 | **8**  **(2%)** |
| **pT1** | 4  (7%) | 11  (8%) | 32  (63%) | 50  (25%) | 7  (17%) | 0 | **104**  **(21%)** |
| **pT2** | 30  (54%) | 47  (35%) | 18  (35%) | 121 (61%) | 22  (55%) | 0 | **238**  **(47%)** |
| **pT3** | 12  (21%) | 43  (32%) | 0 | 18  (9%) | 11  (28%) | 0 | **84**  **(17%)** |
| **pT4** | 6  (11%) | 26  (19%) | 1  (2%) | 0 | 0 | 1  (5%) | **34**  **(7%)** |
| **NA** | 4  (7%) | 8 (6%) | 0 | 2  (1%) | 0 | 1  (5%) | **15**  **(3%)** |
|  |  |  |  |  |  |  |  |
| **FIGO 1** | - | - | - | - | - | 15 (72%) | **15**  **(3%)** |
| **FIGO 2** | - | - | - | - | - | 0 | **0** |
| **FIGO 3** | - | - | - | - | - | 4  (19%) | **4**  **(1%)** |
|  |  |  |  |  |  |  |  |
| **R1** | 1 (  2%) | 0 | 2  (4%) | 24  (12%) | 18  (45%) | 0 | **45**  **(9%)** |
| **pN +** | 18  (32%) | 26 (  19%) | 7  (14%) | 42  (22%) | 8  (20%) | 5  (24%) | **106**  **(21%)** |
|  |  |  |  |  |  |  |  |

*Nr. = number, IQR = interquartile range, BMI = body mass index, SCC = squamous cell carcinoma, MCC = Merkelcel carcinoma, UC= Urethral carcinoma, NA = not answered, c = clinical, p = pathological, T = tumor stage, N= nodal stage, R = resection margin, FIGO = Fédération Internationale de Gynécologie et d'Obstétrique. # Number of procedures is more compared to the included patients in penile cancer patient group, because ~~of~~ part of these patients were treated with a re-sentinel node biopsy procedure, # 6 patients were scheduled for multiple SN procedures, 4 patients were treated with a re-sentinel node and 2 patients were treated with three sentinel node procedures. Patients with prostate cancer presented with >5% risk on LN metastases according to the Briganti nomogram.*

*Injection procedure*

In the patient group with skin malignancies (melanoma, Merkel cell carcinoma (MCC) and Squamous cell carcinoma (SCC)) ICG-^99m^Tc-nanocolloid was intradermally injected in four depots around the primary tumor site (approximately total 90 MBq). In penile cancer, vulvar cancer and oral cavity cancer patients the hybrid tracer was peritumorally injected in three-four depots (approximately total 90 MBq). In prostate cancer patients the hybrid tracer was administered in four depots in the peripheral zone of the prostate (approximately 240 MBq).[1]

*Preoperative imaging procedure*

Dynamic imaging (anterior and lateral) was performed to visualize the drainage of the hybrid tracer via the lymphatic vessels from the injection site in the first ten minutes. Early lymphoscintigraphy was performed 15 minutes after injection of the hybrid tracer. This was repeated at two hours post injection (late lymphoscintigraphy). Planar imaging sessions were performed with a dual-head camera (Symbia T, Siemens, Erlangen, Germany).

Two hours post tracer administration three-dimensional (3D) imaging was performed with SPECT and low-dose CT (40 mAs; 130 kV) (Symbia T) acquisition. SPECT and CT images were fused after correction for scatter and tissue attenuation. SPECT/CT and CT images were correlated using multiplanar reconstruction (Osirix medical imaging software; Pixmeo, Geneva, Switzerland) with volume rendering in order to allow anatomical LN mapping.

To determine the number and anatomical location of the SN(s), the nuclear medicine physician evaluated all acquired images and pointed out the SNs based on the comprehensive use of early and late lymphoscintigraphy as well as fused SPECT/CT images. SNs were identified based on their status of being directly draining LNs for the injection/ primary tumor site.[2] The location of the lymphatic basin containing the SNs was reported to the surgical specialists and the images sent to Picture Achieving and Communication System (PACS) for display in the operating room during SN biopsy. The findings were discussed with the opertatring surgeon, either by the nuclear medicine physician or by the research-associate.

**Surgical procedure**

*Surgical procedure*

In total 501 surgical procedures were evaluated. In short and generalized: when patients were operated in a one-day protocol, the hybrid tracer was injected in the morning of the day of surgery. When patients were operated in a two-day protocol, the injection was performed in the late morning or early afternoon of the day prior to surgery~~.~~ The time-interval between injection and surgery remained < 30 h (Table SI2).

Depending on the indication and the surgeon’s preference, the primary lesion (and thus sites of tracer deposition) could be removed before or after the SN biopsy. This procedural difference was taken into account when analyzing the data. In all patients with melanoma, vulvar and penile cancer, and in a minority (15% (28/186) of patients with head-and-neck cancer (melanoma and oral cancer) (total n = 300) 1.0 mL patent blue V dye (Laboratoire Guerbet, Aulnay-Sous-Bois, France) was administered at the injection sites used for ICG-^99m^Tc-nanocolloid in the operation room, before disinfection. Blue dye was never administered in patients with prostate cancer. In prostate cancer patients, besides SN biopsy an additional extended pelvic lymph node (LN) dissection was performed according to previously described procedures.[1-3]

During surgery a gamma probe, a near-infrared fluorescence camera or near-infrared fluorescence laparoscope and when necessary a portable gamma camera were used to locate, guide and confirm SN removal. Before (*in vivo*) and after (*ex vivo*) resection of the SN, the individual nodes were scored for being radioactive (Y/N), fluorescent (Y/N) and/or blue (Y/N). When possible also the gamma probe counts were registered.

*“Open” surgery*

Surgical procedures were performed by surgical specialists with experience in (radioguided) SN biopsy procedures and experience in the use of blue dye.

For tumors in the head and neck area generally SN biopsy was performed prior to removal of the primary tumor or scar; in these patients radioguided SN identification could be influenced by the radioactivity related to the injections around the primary lesion.

Before incision, a pre-incision overview image of the area harboring the SN was generated with a portable gamma camera (Sentinella, Oncovision, Valencia, Spain) in the head-and-neck area or when SNs were expected in the vicinity of the injection site. Subsequently, a gamma probe (Neoprobe; Johnson & Johnson Medical, Hamburg, Germany), was used to guide the surgeon to the SN using its acoustic read-out.

In the group in which blue dye was used (penile cancer, melanoma, vulva), most surgeons tried to identify the blue lymphatic ducts running to the SN. When the SN was in reach of a couple of mm, a near-infrared fluorescence camera (PhotoDynamic Eye; Hamamatsu Photonics, Hamamatsu, Japan) was used to optically identify the SN harboring the hybrid tracer. To visualize the ICG signal the lights in the operation room had to be dimmed.

After the excision of the SNs the portable gamma camera and fluorescence camera were used to check for residual radio- or fluorescence-activity. When there was still activity left on the spot from where the SN was excised, this indicated that the previously removed SN had been part of a cluster of SNs. These nodes were considered additional SNs and also harvested.

Before closing the SN biopsy wound, it was evaluated for palpable suspicious (non- fluorescent/blue or –radioactive) LN.

*Robot-assisted laparoscopic surgery*

SPECT/CT images were used to identify the area of interest and pin-point anatomical landmarks. After preparation of the tissue, a laparoscopic gamma probe (Europrobe, Strassbourg, France) was introduced via the assistant-portal and was used in similar fashion as in an open procedure, based on the acoustic read out SNs were located. A laparoscopic fluorescence camera (Karl Storz Endoskope GmbH, Tuttlingen, Germany) was introduced via a portal to identify the fluorescence signal within the SNs.[1] After localization of the SNs, an additional ePLND and prostatectomy were performed.

*BMI associated optical detection*

Fatty tissue surrounding the SN could hamper the fluorescence detection of the SN. To study the probable influence of fatty tissue and, as such, body mass index (BMI) we evaluated if fluorescence detection was associated with the BMI of the patient.

*One-day vs. two-day protocol*

Detection rates of the hybrid tracer and blue dye were compared. As the gamma signal is subject to decay, this could hinder the gamma signal detection rate. Here the detection for fluorescence and blue dye were also compared.

**Pathological evaluation**

All LNs identified in the removed tissue specimens entitled SN were examined as SN by an extended pathological protocol. After formalin fixation all SNs were bisected through the central plane and paraffin embedded. Histopathological examination was performed on multiple levels using haematoxylin and eosin staining (H&E) and immunohistochemistry with partly different protocols depending on the cancer type (for further details see below). Immunohistochemistry was performed with an automated IHC system (Ventana BenchMark Ultra, Ventana Medical Systems Inc, Tucson, AZ, USA).

For melanoma the SN was cut at 6 levels with intervals of 50 µm. Each section was stained with H&E and melanA (clone A103, Dako, Carpinteria, CA, USA). For all other cancer types the SN was cut at 3 levels with intervals of 150µm and each section was stained with H&E. Except for vulvar cancer all second levels were used for immunohistochemistry using the following antibodies: CAM (clone CAM5.2, Becton Dickinson, San Jose, CA, USA) for prostate cancer and MCC and pankeratin (clone AE1/3, Thermo Fisher Scientific Inc, Waltham, MA, USA) for penile and oral cancer. All three levels were used for immunohistochemistry for vulvar cancer with the pankeratin clone AE1/3.

*Sentinel node Tumor find rate (_SN_TFR)*

The tumor find rate (TFR) illustrates the rate of probability of finding a tumor-positive SN with SN biopsy. The SN tumor find rate (_SN_TFR) was calculated based on the two following formulas for SPECT/CT and intraoperative SN detection: *positive SNs at pathological evaluation / SN_SPECT/CT_ * 100% =  _SN_TFR_SPECT/CT_* and *positive SNs at pathological evaluation/ SN_intraoperative_ * 100% = _SN_TFR_intraoperative_.*

**Results**

*Preoperative imaging*

On the early- and late lymphoscintigraphy, respectively, 826 and 1209 (31.7% increase) SN-related hotspots were identified. SPECT/CT imaging yielded another 10% increase in SN-related hotspots based on preoperative imaging (1327; Table SI2; Figure SI1). This increase was in line with a previous study and most prominent in the head and neck area and pelvis.[4] By placing the SN-related hot spots within their anatomical context SPECT/CT provided valuable 3D guidance information, a so-called virtual map, for the operating surgeons. SPECT/CT also improved the detection of SNs residing in the proximity of the injection site.

**
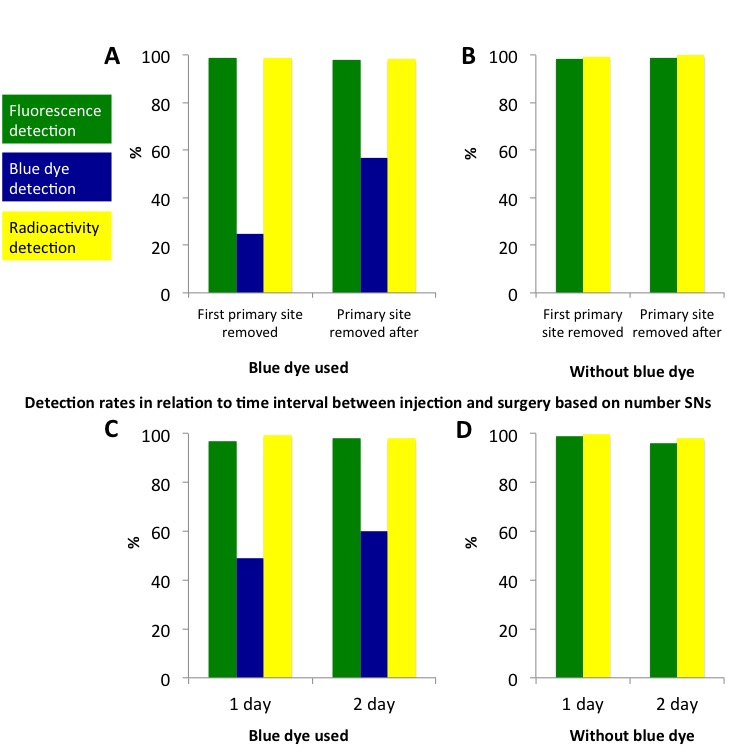
**

**Figure SI1. The influence of the surgical logistics on imaging findings** A) and B) show the detection rates (%) in relation to the order in which the surgical procedure was performed. C) and D) show the detection rates (%) in relation to the time~~s~~ of hybrid tracer administration and the surgical procedure (1- day and 2-day protocol). The green bar represents the fluorescence detection, the blue bar represents the blue optical detection and the yellow bar represents the gamma detection.

**Table SI2. Preoperative imaging findings and postoperative pathological evaluation per indication of primary tumor location**

|  | **Skin malignancies body** | **Head-and-neck skin malignancies** | **Oral cavity** | **Penile cancer** | **Prostate** | **Vulva** | **Total** |
| --- | --- | --- | --- | --- | --- | --- | --- |
| **Injected dose (MBq), median + IQR** | 76  (71-80) | 82  (73-89) | 81  (73-87) | 82  (75-86) | 218  (206-228) | 86  (76-92) | **82**  **(74–90)** |
| **Preoperative imaging findings** |  |  |  |  |  |  |  |
| Total SNs on early lymphoscintigrams | 97 | 272 | 121 | 249 | 51 | 36 | **826** |
| SNs on early lymphoscintigrams,  median (IQR) | 2  (1-2) | 2  (1-3) | 2  (2-3) | 1  (0-2) | 1 (0-2) | 1  (1-2) | **2**  **(1-2)** |
| Total SNs on late lymphoscintigrams | 114 | 318 | 145 | 494 | 89 | 49 | **1209** |
| SNs on late lymphoscintigrams,  median (IQR) | 2  (1-3) | 2  (1-3) | 3  (2-4) | 2  (2-3) | 3 (2-4) | 2  (2-3) | **2**  **(2-3)** |
| Total SNs SPECT/CT | 117 | 348 | 156 | 537 | 119 | 50 | **1327** |
| SNs on SPECT/CT, median (IQR) | 2  (1-3) | 2  (2-3) | 3  (2-4) | 3  (2-3) | 3  (2-4) | 2  (2-3) | **2**  **(2-3)** |
| **Interval between injection and surgery** |  |  |  |  |  |  |  |
| Time (hr) from injection till surgery,  median (IQR) | 18  (5-20) | 5  (5-8) | 5  (4-11) | 6  (5-19) | 5  (4-5) | 7 (4-21) | **5.75**  **(4.57- - 18.53)** |
| Nr. 1-day protocol procedures (%) | 19  (34%) | 104  (78%) | 38  (75%) | 123 (63%) | 40 (100%) | 12 (57%) | **336**  **(68%)** |
| Nr 2-day protocol procedures (%) | 37  (66%) | 30  (22%) | 13  (25%) | 72 (37%) | 0 | 9 (43%) | **161**  **(32%)** |
| **Post-operative pathology findings** |  |  |  |  |  |  |  |
| Total SNs detected at pathology | 138 | 583 | 293 | 691 | 160 | 73 | **1938** |
| Total LNs* detected at pathology | 155 | 656 | 357 | 891 | 326 | 89 | **2474** |
| Total nr. positive SNs | 24 | 35 | 10 | 41 | 16 | 6 | **132** |

*MBq = MegaBequerel, IQR = Interquartile range, SN = sentinel node, LN = lymph node, nr. = number,. * These are all nodes find after pathological evaluation, sentinel nodes and LNs together. In the penile cancer patient group and in he prostate cancer group relatively more LNs were found because an additional LN dissection.*

*Intraoperative SN identification vs. SPECT/CT SN identification*

Preoperative imaging with SPECT/CT suffers from its inability to accurately define clustered SNs and can thus underestimate the exact number of SNs that require resection.[1-5] This combined with the improved surgical guidance realized resulted in a discrepancy between preoperatively defined SN-hotspots and excised SN specimens (1327 SNs vs. 1643 SNs; Table SI1, Figure SI1), which in some case lead to prolongation of the surgical procedures. Here it is important to mention that the increased nodal resection did not negatively influence the morbidity rate when compared to earlier studies using mono-tracers.[6-10] The influence of the increased nodal resection in relation to the tumor find rate is discussed below.

As also reported earlier [6-11], prospective analysis of the CT scans indicated that SN-related hot-spots as observed on SPECT in some cases overlapped with multiple LNs. Recent findings suggest that D-prep MRI could in the future help improve delineation of individual LNs within a single hot-spot.[11]

*Association with body mass index detection rates*

Body mass index (BMI) negatively was shown to influence the *in vivo* fluorescence guidance the radioguidance procedure (Figure SI2). The increase in detection probability for gamma tracing, which was not statistically significant *in vivo* (p=0.87) nor *ex vivo* (p=0.41), can be partly explained by the fact that in patients with a higher BMI surgeons automatically rely more on this technology than on the optical alternatives. More careful *ex vivo* imaging of the resected tissues yielded improved fluorescence-based identification rates, thereby indicating that this shortcoming was the result of signal attenuation caused by fatty tissues overlaying the SNs. The blue dye identification also improved following *ex vivo* tissue examination.

**
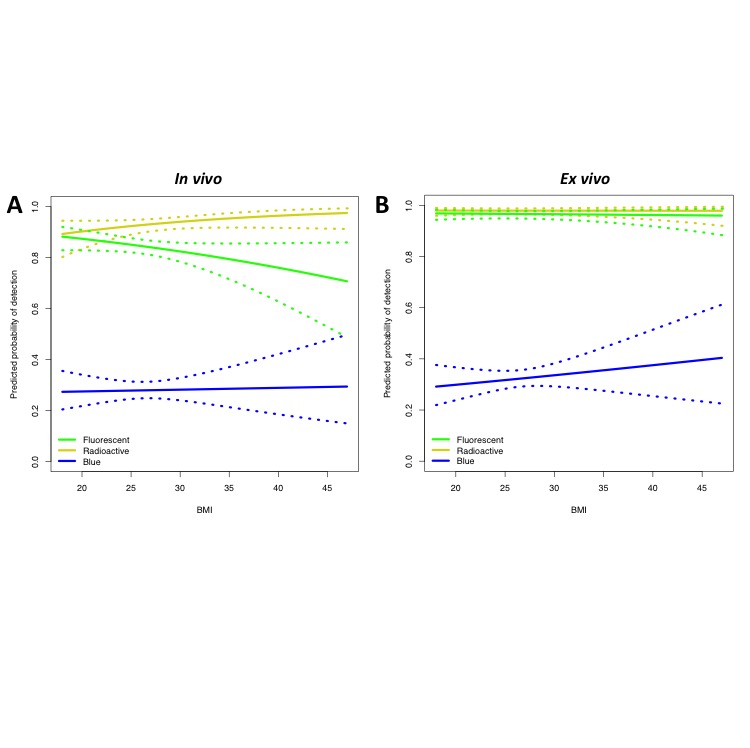
**

**Figure SI2. Detection curves in relation to body mass index (BMI)** A) Depicts the relation between body mass index (BMI) and the *in vivo* probability of detection for the respective modalities used. B) Depicts the same feature, but here the additional *ex vivo* screening was used to extend the *in vivo* findings. The broken lines represent the 95% confidence interval.

*One-day vs. two-day protocol*

In the surgical community there is debate with regard to the order wherein SN procedures are performed (primary tumor site removal followed by SN biopsy or SN biopsy followed by primary tumor site removal, or one-day vs. two-day protocol).[12] We found that the order in which SN biopsy and primary tumor removal was performed did not influence the detection rates of the hybrid tracer (>95% based on *ex vivo* validation). It also indicates that the hybrid tracer reaches its optimal drainage pattern prior to surgery (>2h). However, there was an association between the order, and the blue dye-based SN identification rates: more blue nodes were visualized in patients wherein the SN biopsy was performed before primary tumor removal (p<0.0001) (Figure SI1). This indicates that migration of lymphangiographic agents such as blue dye to the SNs still occurs during the resection process.[13]

No significant differences in fluorescence- or radioactivity-based SN detection percentages between the one- and two-day protocols were observed (Figure SI1 C, D). This finding indicates that migration of lymphangiographic agents such as blue dye to the SNs still occurs during the resection process.[13]

*Sentinel node Tumor find rate (_SN_TFR)*

_SN_TFR rates were used to place the additionally resected SNs in clinical perspective by relating the number of resected SNs to the 132 tumor positive SNs. Hereby we compensate for the fact that all the SNs identified at pathology are harvested of the surgically dissected specimens labeled as SN. This gave an _SN_TFR_intraoperative_ of 8% (132/1643), with a 5.2-18.3% range based on the indication for SN biopsy this decreased slightly if we compensate for the SNs that were not removed 7.8% (132/1693). When the same number was related to the SN-associated hotspots preoperatively identified by SPECT/CT, the _SN_TFR_SPECT/CT_ was 9.9 % (132/1327).

Using the _SN_TFR values we could show that an overall increase of 19.2 % (611 SNs) resected SNs compared to those identified on SPECT/CT, only yielded a non-proportional 2.1% drop in _SN_TFR (Table SI3). If the resection of these additional SNs had no oncologic value, i.e. the additional nodes did not contain cancer, it would be reasonable to expect this drop would have been more substantial. Hence, not resecting these additional SNs could potentially have left nodal metastasis in situ, which may have impacted the FN rates.[6, 8, 10] Since all LNs in the SN labeled pathological specimens were deemed SNs, this gave a 15% increase in SNs-rate compared to the amount of SNs identified during surgery. This increase complicated pathological analysis and was by far the highest in the head-and-neck area, where a 34% increase was presented (Table SI1).

**Table SI3. Intraoperative detection per location**

| **Intraoperative detection per location *in vivo* detection** | | | | | | | | | |
| --- | --- | --- | --- | --- | --- | --- | --- | --- | --- |
|  | **Head-and-neck** | **Shoulder/arm** | | **Axilla** | **Inguinal** | **Pelvic area** | | **Other** | **Total** |
| ***Sentinel node evaluated with blue dye*** | | | | | | | | | |
| **No optical identification (SNs)** | 1  (1%) | 0 | | 2  (3%) | 11  (2%) | - | | 0 | 14 |
| **Blue only SNs** | 0 | 0 | | 1  (1%) | 6  (1%) | - | | 0 | 7 |
| **Fluorescent only SNs** | 80  (76%) | 4  (80%) | | 22  (29%) | 270  (43%) | - | | 1  (50%) | 377 |
| **Fluorescent and blue SNs** | 24  (23%) | 1  (20%) | | 52  (67%) | 340  (54%) | - | | 1  (50%) | 418 |
| **Total fluorescent SNs** | 104  (99%) | 5  (100%) | | 74  (96%) | 610  (97%) |  | | 100 (100%) | 893  (98%) |
|  |  |  | |  |  |  | |  |  |
| **SNs not evaluated for staining** | 13 | 0 | | 3 | 117 | - | | 0 | 133 |
|  |  |  | |  |  |  | |  |  |
| **Radioactive SNs** | 117  (99%) | 5  (100%) | | 80  (100%) | 722  (97%) | - | | 2 (100%) | 926 (98%) |
| **SNs NA for radioactivity** | 0 | 0 | | 0 | 0 | 0 | | 0 | 0 |
|  |  |  | |  |  |  | |  |  |
| ***Sentinel nodes evaluated without blue dye*** | | | | | | | | | |
| **No optical identification (SNs)** | 22  (4%) | - | | - | - | 28  (22%) | | - | 50  (8%) |
| **Fluorescent SNs** | 482  (96%) | - | | - | - | 99 (78%) | | - | 581  (92%) |
| **SNs not evaluated for staining** | *61* | *-* | | *-* | *-* | *0* | | *-* | *61* |
|  |  |  | |  |  |  | |  |  |
| **Radioactive SNs** | 498  (96%) | - | | - | - | 82 (100%) | | - | 580 (97%) |
| **SNs not evaluated for radioactivity** | 0 | - | | - | - | 0 | | - | 0 |
|  |  |  | |  |  |  | |  |  |
| **Detection per location (*ex vivo* and *in vivo* combined)** | | | | | | | | | |
|  | **Head-and -neck** | | **Shoulder/arm** | **Axilla** | **Inguinal** | | **Pelvic area** | **Other** | **Total** |
| ***Sentinel node evaluated with blue dye*** | | | | | | | | | |
| **Not stained SNs** | 1  (1%) | | 0 | 0 | 6  (1%) | | - | 0 | 7  (1%) |
| **Blue SNs** | 0 | | 0 | 0 | 9 (1%) | | - | 0 | 10  (1%) |
| **Fluorescent SNs** | 90  (77%) | | 4  (80%) | 22  (27%) | 310  (43%) | | - | 1  (50%) | 427 (46%) |
| **Fluorescent and blue SNs** | 26  (22%) | | 1  (20%) | 58  (73%) | 397  (55%) | | - | 1  (50%) | 483 (52%) |
| **Total Fluorescent** | 116  (99%) | | 5  (100%) | 80  (100%) | 707  (98%) | |  | 2 (100%) | 910 (98%) |
|  |  | |  |  |  | |  |  |  |
| ***SNs not evaluated for staining*** | 0 | | 0 | 0 | - | | - | 0 | 0 |
|  |  | |  |  |  | |  |  |  |
| **Radioactive SNs** | 118  (100%) | | 5  (100%) | 80  (100%) | 726  (98%) | | - | 2 (100%) | 931 (99%) |
|  |  | |  |  |  | |  |  |  |
| ***Sentinel nodes evaluated without blue dye*** | | | | | | | | | |
| **Not stained SNs** | 6  (1%) | | - | - | - | | 1  (1%) | - | 7  (1%) |
| **Fluorescent SNs** | 552  (99%) | | - | - | - | | 126  (99%) | - | 678 (99%) |
|  |  | |  |  |  | |  |  |  |
| **SNs not evaluated for staining** | *8* | | *-* | *-* | *-* | | *0* | *-* | *8* |
|  |  | |  |  |  | |  |  |  |
| **Radioactive** | 553  (99%) | | - | - | - | | 127  (100%) | - | 680 (99%) |
| **SNs not evaluated for radioactivity** | 0 | | - | - | - | | 0 | - | 0 |

*Overall survival*

In the Kaplan Meier curve of the total study population, with a median follow-up of 33 months, a 78% (CI 67%-90%) survival was found (Figure SI3). The overall 5-year survival for the different indications ranged from 40% to 90%, dependent on the primary tumor type (Figure SI3B). In all the groups of pR0 patients (n= 437 (88%)), the overall survival decreased when patients were staged pN+ after SN biopsy (Figure SI3 C, D).

Despite the fact that the individual patient groups still were relatively small, from overall survival curves can be deduced that the survival for the prostate cancer patients (n = 40; these patients also received an extensive pelvic nodal dissection in addition to the SN procedure), penile cancer patients (n = 186) and the head-and-neck melanoma group (n = 128) were the lowest. Higher survival rates were found for the patients with a melanoma located on the body (n = 53), and the vulva cancer patients (n =19). Unfortunately, the evaluated groups were too small to allow further stratification of the tumors. Stratification did show that smaller tumors without pN+ have a longer overall survival compared to the higher T-stage tumors. These survival curves are comparable to the ones previously reported for SN procedures performed using routine radioguidance procedures.[10, 14-16] For prostate cancer patients after SN biopsy and ePLND combined with prostatectomy no literature on survival rates is available, as has been described by Wit et al.[3]


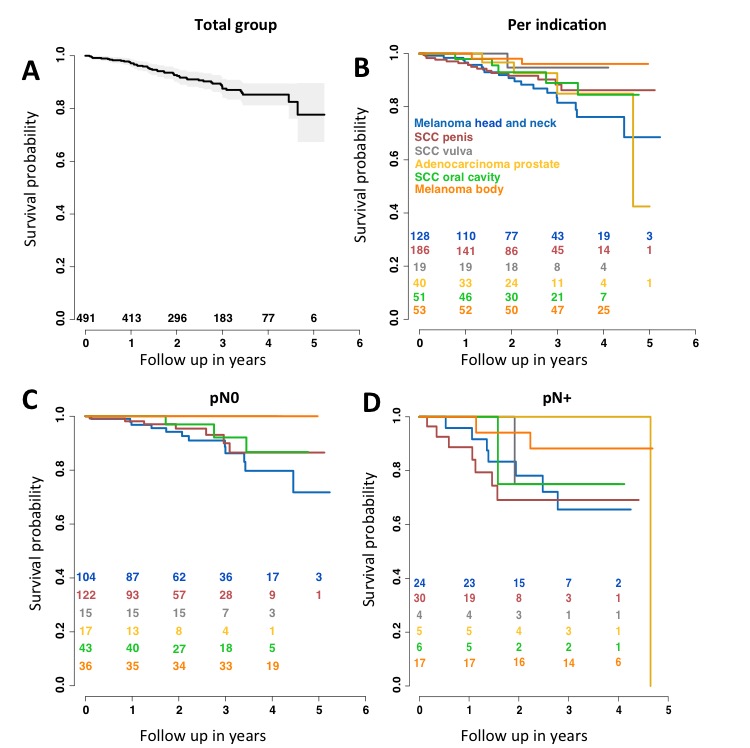


**Figure SI3. Overall survival curves:** A) The overall Kaplan Meier survival curve of all patients combined, B) The overall survival for each individual group. In C) and D) the survival curves are shown for the pN0 and pN+ patients, respectively.

**Table SI4. Complication rates (Clavien-Dindo), TFR, False negative rates.**

| **Complications rate**  **(Clavien dindo)** | **Melanoma body** | **Head and neck melanoma** | **Oral cavity cancer** | **Penile cancer** | **Prostate cancer** | **Vulva cancer** | **Total** |
| --- | --- | --- | --- | --- | --- | --- | --- |
|  |  |  |  |  |  |  |  |
| **Grade I** | - | 3  (2%) | 2  (4%) | 4  (2%) | 1  (3%) | 2 (10%) | **12**  **(2.4%)** |
| **Grade II** | - | 3  (2%) | 2  (4%) | 21  (11%) | 5  (13%) | 1 (5%) | **32**  **(6.4%)** |
| **Grade IIIa** | - | - | - | 6  (3%) | 3  (8%) | 1 (5%) | **10**  **(2.0%)** |
| **Grade IIIb** | 1  (2%) | 1  (1%) | 1  (2%) | 6  (3%) | 1  (3%) | - | **10**  **(2.0%)** |
| **Grade IVa** | - | 1  (1%) | - | 2  (1%) | - | - | **3**  **(0.6%)** |
| **Grade IVb** | - | - | - | - | - | - | **-** |
| **Grade V** | - | - | - | 1  (1%) | - | - | **1**  **(0.2%)** |
|  |  |  |  |  |  |  |  |
|  |  |  |  |  |  |  |  |
| **_SN_TFR_SPECT/CT_** | 20.5%  (24/117) | 10.1%  (35/348) | 6.4%  (10/156) | 7.6% (41/537) | 13.4% (16/119) | 12% (6/50) | **9.9% (132/1327)** |
| **_SN_TFR_intraoperative_** | 18.3%  (24/131) | 7.3%  (35/482) | 5.2%  (10/193) | 6.4%  (41/640) | 12.6% (16/127) | 9.7% (6/62) | **8.0% (132/1643)** |
|  |  |  |  |  |  |  |  |
| **False negative rate**  **(FNR in %)** | 0% | 7%  (2/28) | 22%  (2/9) | 16%  (6/37)^$^ | 13  (1/8) | 0% | **14% (12/87)** |
| **FNR**  **without initial n=15** | 0% | 7%  (2/28) | 0% | 16%  (6/37) | * | # | **10.1%**  **(8/ 79)** |
| **FNR**  **based on groins** | - | - | - | 15%  (6/40) | - | 0% | **-** |
| **FNR without**  **re-sentinel nodes** | - | - | - | 14%  (5/36) | - | 0% | **-** |

TFR = tumor find rate; FNR = false negative rate; ^$^ FNR 19% including pN1 patient; * previous reported patients not analyzed for this study, # only 19 patients analyzed.

**References**

1. KleinJan, G.H., et al., *Optimisation of fluorescence guidance during robot-assisted laparoscopic sentinel node biopsy for prostate cancer.* Eur Urol, 2014. 66(6):991-8.

2. Nieweg, O.E., P.J. Tanis, and B.B. Kroon, *The definition of a sentinel node.* Ann Surg Oncol, 2001. **8**(6):538-41.

3. Wit, E.M.K., et al., *Sentinel Node Procedure in Prostate Cancer: A Systematic Review to Assess Diagnostic Accuracy.* Eur Urol, 2017. 71(4):596-605.

4. Jimenez-Heffernan, A., et al., *Results of a Prospective Multicenter International Atomic Energy Agency Sentinel Node Trial on the Value of SPECT/CT Over Planar Imaging in Various Malignancies.* J Nucl Med, 2015. 56(9):1338-44.

5. van den Berg, N.S., et al., *Multimodal Surgical Guidance during Sentinel Node Biopsy for Melanoma: Combined Gamma Tracing and Fluorescence Imaging of the Sentinel Node through Use of the Hybrid Tracer Indocyanine Green-(99m)Tc-Nanocolloid.* Radiology, 2015. 275(2):521-9.

6. Flach, G.B., et al., *Sentinel lymph node biopsy in clinically N0 T1-T2 staged oral cancer: the Dutch multicenter trial.* Oral Oncol, 2014. 50(10):1020-4.

7. Kretschmer, L., et al., *Nodal Basin Recurrence After Sentinel Lymph Node Biopsy for Melanoma: A Retrospective Multicenter Study in 2653 Patients.* Medicine (Baltimore), 2015. 94(36):e1433.

8. Pezier, T., et al., *Sentinel lymph node biopsy for T1/T2 oral cavity squamous cell carcinoma--a prospective case series.* Ann Surg Oncol, 2012. 19(11):3528-33.

9. Valsecchi, M.E., et al., *Lymphatic mapping and sentinel lymph node biopsy in patients with melanoma: a meta-analysis.* J Clin Oncol, 2011. 29(11):1479-87.

10. Van der Zee, A.G., et al., *Sentinel node dissection is safe in the treatment of early-stage vulvar cancer.* J Clin Oncol, 2008. 26(6):884-9.

11. Buckle, T., et al., *Diffusion-weighted-preparation (D-prep) MRI as a future extension of SPECT/CT based surgical planning for sentinel node procedures in the head and neck area?* Oral Oncol, 2016. 60:48-54.

12. Dimopoulos, P., et al., *Dynamic sentinel lymph node biopsy for penile cancer: a comparison between 1- and 2-day protocols.* BJU Int, 2016. 117(6):890-6.

13. Manny, T.B., M. Patel, and A.K. Hemal, *Fluorescence-enhanced robotic radical prostatectomy using real-time lymphangiography and tissue marking with percutaneous injection of unconjugated indocyanine green: the initial clinical experience in 50 patients.* Eur Urol, 2014. 65(6):1162-8.

14. Djajadiningrat, R.S., et al., *Contemporary management of regional nodes in penile cancer-improvement of survival?* J Urol, 2014. 191(1):68-73.

15. Lee, D.Y., et al., *Predictors and Survival Impact of False-Negative Sentinel Nodes in Melanoma.* Ann Surg Oncol, 2016. 23(3):1012-8.

16. Schilling, C., et al., *Sentinel European Node Trial (SENT): 3-year results of sentinel node biopsy in oral cancer.* European Journal of Cancer. 51(18):2777-2784.
